# Supplementary material for: Saccharomyces boulardii (CNCM I-745) improves intestinal damage in sepsis by remodeling intestinal flora structure
Source: Front Cell Infect Microbiol. 2025 Jul 31;15:1602792. doi: 10.3389/fcimb.2025.1602792 (PMC12350329; doi:10.3389/fcimb.2025.1602792)
Supplement: Supplementary file 1 [file DataSheet1.zip › summary/summary/7_Differential_analysis/2_LEfSe_result/Group_BW4_vs_AW4_0.05_3.0/LEfSe_Group_BW4_vs_AW4.abund.pdf]

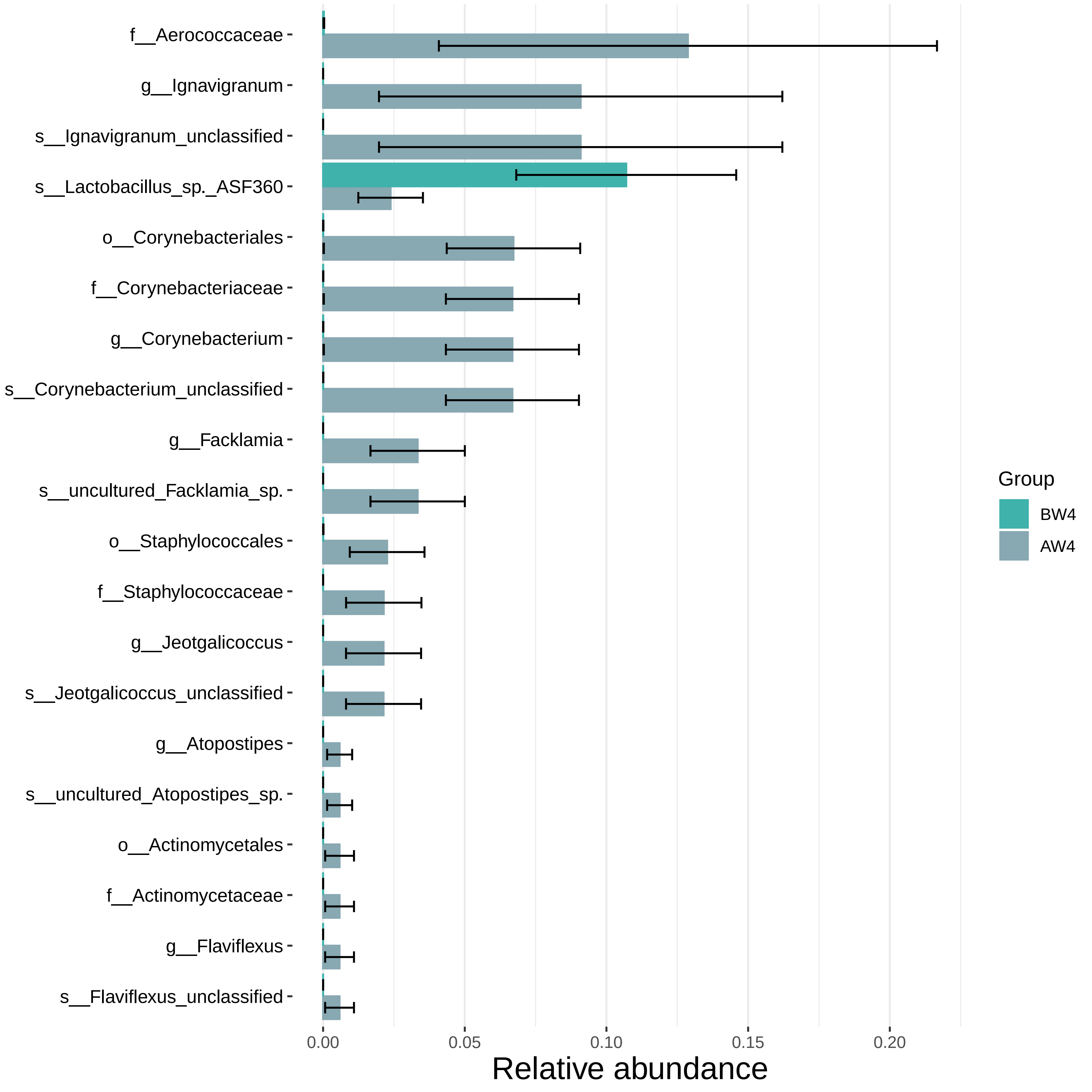
f__Aerococcaceae
g__Ignavigranum
s__Ignavigranum_unclassified
s__Lactobacillus_sp._ASF360
o__Corynebacteriales
f__Corynebacteriaceae
g__Corynebacterium
s__Corynebacterium_unclassified
g__Facklamia
s__uncultured_Facklamia_sp.
o__Staphylococcales
f__Staphylococcaceae
g__Jeotgalicoccus
s__Jeotgalicoccus_unclassified
g__Atopostipes
s__uncultured_Atopostipes_sp.
o__Actinomycetales
f__Actinomycetaceae
g__Flaviflexus
s__Flaviflexus_unclassified
0.00
0.05
0.10
0.15
0.20
Relative abundance
Group
BW4
AW4
